# Predicting health-related social needs in Medicaid and Medicare populations using machine learning

**DOI:** 10.1038/s41598-022-08344-4

**Published:** 2022-03-16

**Authors:** Jennifer Holcomb, Luis C. Oliveira, Linda Highfield, Kevin O. Hwang, Luca Giancardo, Elmer Victor Bernstam

**Affiliations:** 1 Department of Management, Policy, and Community Health, The University of Texas Health Science Center at Houston (UTHealth) School of Public Health, 1200 Pressler St, Houston, TX 77030 USA; 2 Sinai Urban Health Institute, 1500 South Fairfield Avenue, Chicago, IL 60608 USA; 3 The University of Texas Health Science Center at Houston (UTHealth) School of Biomedical Informatics, 7000 Fannin, Houston, TX 77030 USA; 4 Houston Methodist Academic Institute, 6670 Bertner Ave, Houston, TX 77030 USA; 5 Departments of Management, Policy, and Community Health and Epidemiology, Human Genetics and Environmental Sciences, The University of Texas Health Science Center at Houston (UTHealth) School of Public Health, 1200 Pressler St, Houston, TX 77030 USA; 6 Department of Internal Medicine, The University of Texas Health Science Center at Houston (UTHealth) John P and Katherine G McGovern Medical School, 6410 Fannin, Houston, TX 77030 USA; 7 Center for Healthcare Quality and Safety at UTHealth/Memorial Hermann, The University of Texas Health Science Center at Houston (UTHealth) John P and Katherine G McGovern Medical School, 6410 Fannin, Houston, TX 77030 USA; 8 Center for Precision Health, The University of Texas Health Science Center at Houston (UTHealth) School of Biomedical Informatics, 7000 Fannin, Houston, TX 77030 USA

**Keywords:** Population screening, Computer science

## Abstract

Providers currently rely on universal screening to identify health-related social needs (HRSNs). Predicting HRSNs using EHR and community-level data could be more efficient and less resource intensive. Using machine learning models, we evaluated the predictive performance of HRSN status from EHR and community-level social determinants of health (SDOH) data for Medicare and Medicaid beneficiaries participating in the Accountable Health Communities Model. We hypothesized that Medicaid insurance coverage would predict HRSN status. All models significantly outperformed the baseline Medicaid hypothesis. AUCs ranged from 0.59 to 0.68. The top performance (AUC = 0.68 CI 0.66–0.70) was achieved by the “any HRSNs” outcome, which is the most useful for screening prioritization. Community-level SDOH features had lower predictive performance than EHR features. Machine learning models can be used to prioritize patients for screening. However, screening only patients identified by our current model(s) would miss many patients. Future studies are warranted to optimize prediction of HRSNs.

## Introduction

The association of social determinants of health (SDOHs) and social needs with health outcomes has been recognized internationally and in the United States. While often used interchangeably, these are distinct concepts. SDOHs are broader upstream social conditions in which people are born, live, and work while social needs are more immediate and downstream individual or family needs impacted by the conditions^1,2^. Social needs such as food insecurity have been associated with depression^3^, diabetes distress^3^, and chronic health conditions^4–7^. Similarly, children who experience energy insecurity (i.e., inability to obtain energy to heat or cool one’s home) in their household are at an increased odds of food insecurity, hospitalization, and poor health^8^. Unmet social needs have also been associated with missed medical appointments, more frequent emergency department (ED) use and hospital readmission^9,10^. There is increasing evidence of the impact of social interventions to increase access to preventive healthcare^11^, improve management of chronic conditions^11^, and reduce hospital admissions^12,13^, reducing healthcare costs.^14–16^.

To achieve more equitable health outcomes at lower costs^17^, healthcare systems should prioritize individual patients for social interventions^18^. Screening patients, particularly those who are low-income and those at highest risk for adverse health outcomes, is an important step in addressing social needs^19^. Current approaches to screening for social needs in U.S. healthcare settings rely on universal screening of patients. Various universal screening approaches have been tested through the Protocol for Responding to and Assessing Patient Assets, Risks, and Experiences (PRAPARE)^20^, Your Current Life Situation screening tool developed by the Kaiser Permanente Care Management Institute^21^, and the Accountable Health Communities (AHC) Model screening tool developed by the Centers for Medicare & Medicaid Services (CMS) Innovation Center (CMMI)^22,23^. The PRAPARE social needs assessment has been used frequently across healthcare settings and aligns with national data systems (e.g., Uniform Data System used by the Health Resources and Services Administration with Federally-Qualified Community Health Centers (FQHCs)^24^. A pilot approach to universal health-related social need (HRSN) screening through CMMI’s AHC Model^22,23^ is currently being implemented by 28 organizations across the U.S. However, the U.S. currently lacks standards and guidelines related to the collection of social needs screening data, particularly in healthcare settings^25–28^. Surveying patients requires healthcare staff to build trust with patients and for healthcare systems to increase healthcare spending to ensure dedicated healthcare staff, electronic health record (EHR) infrastructure, other resources (e.g., funding, staff training, screening materials) and time^29–31^. Additionally, studies have shown that healthcare staff, including primary care physicians, do not feel confident screening for and responding to social needs, leading to low screening rates^30,32^. Integration of HRSN data into the EHR in an actionable format continues to present a challenge for healthcare providers and limits screening as a pathway to addressing social needs^33^.

An alternative approach to universal screening is to utilize patient risk scores or risk prediction models to identify and prioritize patients who are most likely to have HRSNs. Risk scores are already widely used in healthcare settings to predict a range of outcomes from specific disease conditions (e.g., cardiovascular disease) to hospital readmissions, healthcare cost, and ED utilization^34–38^. Recently, there has been increasing interest in using SDOH and social needs data to improve risk prediction models. Risk prediction efforts linking community-level geocoded data with EHRs and other patient-level data sources (e.g., claims/administrative data) are nascent and to date have primarily focused on predicting healthcare utilization, such as hospital readmission and ED visits^34,39–41^. Studies have been limited by lack of data on individual level social needs and in most cases limiting to a single healthcare provider or system^34,41,42^. To date, few studies attempted to predict individual patient social needs^43^. These studies attempted to predict social service referrals rather than whether the patient reported a social need.

An opportunity exists to better understand the potential for integrating risk prediction to proactively identify patients in need of further social need assessment or social intervention outside of the healthcare model. Predicting HRSN status is a novel application of predictive models and highly relevant and actionable as screening is the first step in the social intervention pathway. Risk prediction could also help address the structural and logistical barriers to universal HRSN screening implementation that have been identified in recent research, including the low level of uptake by providers, lack of time, EHR integration, availability of trained or skilled staff to conduct screening (and intervention), patient preference, and increased costs, which are often not reimbursed^22–26^. To our knowledge, there are not currently studies available comparing universal versus targeted screening approaches for HRSN. However, research in other health domains such as HIV indicates that targeted screening can be beneficial when implementation barriers such as those noted above are present^44^. The objective of this study was to predict HRSNs of patients in the CMMI AHC Model from patient-level EHR data and publicly available community-level SDOH data. We evaluated the predictive performance versus a baseline method using Medicaid status to assume existence of HRSNs. Our hypothesis was that using a combined dataset would outperform any single data source alone. We further hypothesized that patients insured by Medicaid would be likely to have a HRSN and that the combined dataset would more accurately predict social needs status (e.g., positive or negative) than the Medicaid assumption.

## Methods

### Study design

Patient-level HRSN screening data were collected from September 2018 through December 2020 in the Greater Houston area, Texas in a cross-sectional study design. The AHC Model implementation in the Greater Houston area is a part of a national randomized controlled trial funded by CMMI to test a systematic approach to HRSN screening, community resource referral, and community resource navigation of CMS beneficiaries^22,23,45^. Any community-dwelling CMS beneficiaries accessing care across 13 clinical delivery sites including Emergency Departments (ED), Labor and Delivery Departments and ambulatory clinics in three large health systems were eligible to be screened. The three health systems included a nonprofit, private hospital system (Health System A), a network of outpatient clinics at an academic health university (Health System B), and a safety net hospital (Health System C). Patient EHR data and community-level SDOH data were combined to predict the HRSN status of those patients in the AHC Model. Eligible patients for analysis were those with a completed screening survey in the AHC Model, EHR data from two years prior to HRSN screening date, and an address for geocoding to facilitate linkage of community-level SDOH data. This study has been approved by the Committee for the Protection of Human Subjects (CPHS, the UTHSC‐H Institutional Review Board) under protocol HSC‐SBMI‐13‐0549. All methods were performed in accordance with the relevant guidelines and regulations. Informed consent for this study was waived by the CPHS as part of protocol HSC-SBMI-13-0549.

### Data features

Individual patient-level EHR data included demographics, diagnosis codes (ICD-10), procedure codes (CPT codes for ambulatory and hospital), and insurance type (Medicare, Medicaid or dually covered). For ICD-10 codes, only part of the code describing the disease category was used in order to create clinically relevant "clusters". For community-level SDOH features, we reviewed the existing literature to identify potential SDOH factors associated with known health outcomes, healthcare utilization, and HRSNs^13,19,40,46–50^. Community-level SDOH data at the Texas state Census Tract level were derived from the 5-year (2015 to 2019) estimates from U.S. Census Bureau’s American Community Survey (ACS) and the Centers for Disease Control and Prevention’s Social Vulnerability Index (SVI) (2018). The 12 SDOH features include median household income, poverty level, educational attainment, unemployment, health insurance coverage including uninsured and public insurance, car ownership, home ownership, Supplemental Nutrition Assistance Program (SNAP) benefits, overcrowding, and disability from the ACS and from the SVI, minority status and language. A description of the EHR and Census data can be found in the Supplementary Material.

As part of the HRSN survey patients were asked about four indicators of social needs, and for this study, we developed models predicting the need for each of these indicators based on a patient’s EHR and associated ACS and SVI Census data^23^. We used the following indicators based on 4 of the 5 core social needs screened for in the AHC Model^23^: (1) Core need: housing situation—Identifies whether respondent has HRSN related to housing stability and/or housing quality. (2) Core need: food insecurity—Identifies whether respondent has HRSN related to purchasing food. (3) Core need: transportation—Identifies whether respondent has HRSN related to accessing reliable transportation. (4) Core need: utilities—Identifies whether respondent has HRSN related to difficulty paying utility bills. (5) Any core need—This indicator is true if at least one of the four core needs is true. (6) All core needs—This indicator is true if all of the four core needs are true. In addition to these metrics, we used the Medicaid ID on the survey to indicate whether the respondent was a Medicaid beneficiary. This metric was used as a baseline for testing the predictive model, under the assumption that respondents who are Medicaid beneficiaries would have HRSNs.

### Data linkage

**Figure 1:**
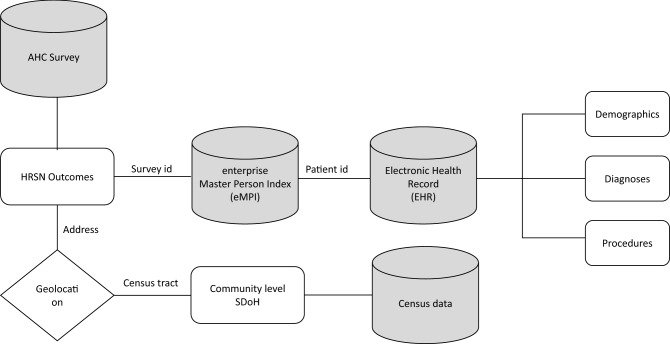
Data sources and linkage for modeling. Flow chart showing the data sources combined to create the dataset displayed. The data sources are displayed as three cylinders displaying the data linking between sources. The measures from each source are displayed as a rectangle linking to other cylinders. Patient-level HRSNs were collected in the AHC screening survey. Using survey demographic data, patients were mapped using a Master Patient Index database (MPI) to patient ID, demographics, diagnosis, and procedures in the EHR. Patients addresses provided in the survey and the EHR were geocoded to each patient’s address and corresponding Census tract. The geocoding is displayed as a diamond connected to the HRSN survey data measures.

**Figure 2:**
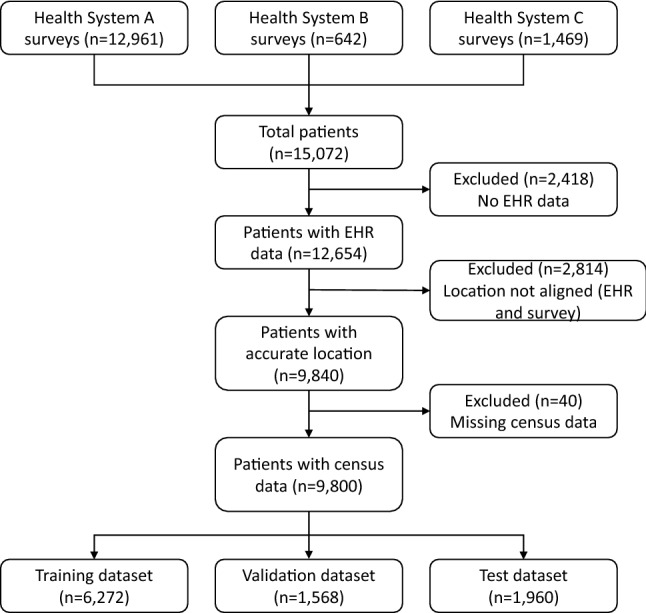
Consort flow diagram. Consort flow diagram depicting the sample size at each step of the data linkage. The diagram moves downward with each step displayed as a rectangle. Patients were excluded from the final datasets if they had no EHR data, if there was not alignment with the EHR and survey addresses, and if their geocoded location was missing corresponding Census data. These exclusions are depicted as rectangles with arrows along the diagram indicating where a patient sample was excluded. From these exclusions, the bottom and final three rectangles depict the training, validation, and test datasets included in the data analysis.

Figure 1 illustrates how the data sources were combined to create the combined dataset. We used a table specially created in the Master Patient Index database (MPI) to map patient IDs from the HRSN survey to the corresponding patient ID in the EHRs^51^. The Match Analysis Methodology in the MPI uses key information from the HRSN surveys like survey patient ID, first name, last name, middle name, date of birth, sex, address (city, state, zip) Medicare Beneficiary Identifier (Medicare), Medicare effective date, and Medicaid effective date to link the records to the EHR data. We used the address provided in the survey and the EHR to geocode each patient’s address and then determined the corresponding Census tract for the address. At each stage of matching, exclusion criteria were applied. HRSN surveys without corresponding EHR data for the patient were excluded (n = 2418). Any records whose geocode did not match between the HRSN survey data and the EHR data as were excluded (n = 2814). These records had a greater than 1-km difference between the address provided in the HRSN survey and in the EHR. The corresponding Census Tract was then used to match the SDOH information from Census data. Any records matched with Census Tracts located outside of the state of Texas were excluded because they could not be matched with SDOH information (n = 40). A Consort flow diagram^52^ was used to depict sample size at each step in Fig. 2.

### Data analysis

First, we randomly allocated the samples into three datasets: 20% of the samples (n = 1960) were allocated for the test set (not used during the training process); 80% of the remaining samples (n = 6272) were allocated for the training set (64% of the entire data set), with the remaining samples (n = 1568) allocated for the validation set (16% of the entire data set. These datasets were stratified such that each group contained approximately the same percentage of samples of each target class as the complete set. The test set was reserved for testing and was neither used for model training, nor for manually or automatically evaluating feature selection or any of the other model parameters. A Gradient Boosting Decision Tree Machine Learning algorithm (LightGBM) was used to predict HRSN status using the individual and combined data sets^53^. These types of algorithms offer some degree of interpretability and work particularly well in machine learning problems with a high dimensionality, large number of features, and large sparsity of data, which is one of the main hurdles when dealing with EHR data. LightGBM is inherently able to handle missing data which allows us to avoid any type of artificial data imputation. The LightGBM model hyperparameters were tuned using a Bayesian optimization, which allowed for an unbiased search of the best performing model without direct trial and error which could lead to overfitting^54^. Specifically, we used the scikit-optimize library^55^ for a crossed validated Bayesian search (implemented in the BayesianSearchCV scikit-optimize class) on the training set. The best combination of hyperparameters was selected by maximizing the accuracy on the validation set. The test set remained completely independent from the hyperparameter search, thereby avoiding any risk of overfitting and data leakage. For a full description of LightGBM and the Bayesian hyperparameter search we refer the readers to the papers referenced^53–55^.

Area Under the Receiving Operating Characteristic Curve (AUC)^56^ and comparison to a baseline decision using Medicaid status were used to evaluate model performance on the test set. Analysis was performed using the Python scikit-learn and lightGBM libraries. P-values were also computed with a non-parametric Mann–Whitney U test, under the null hypothesis that, for each HRSN, the distribution of the ordinal real value output of the models is equal when HRSN = False or HRSN = True.

## Results

**Figure 3:**
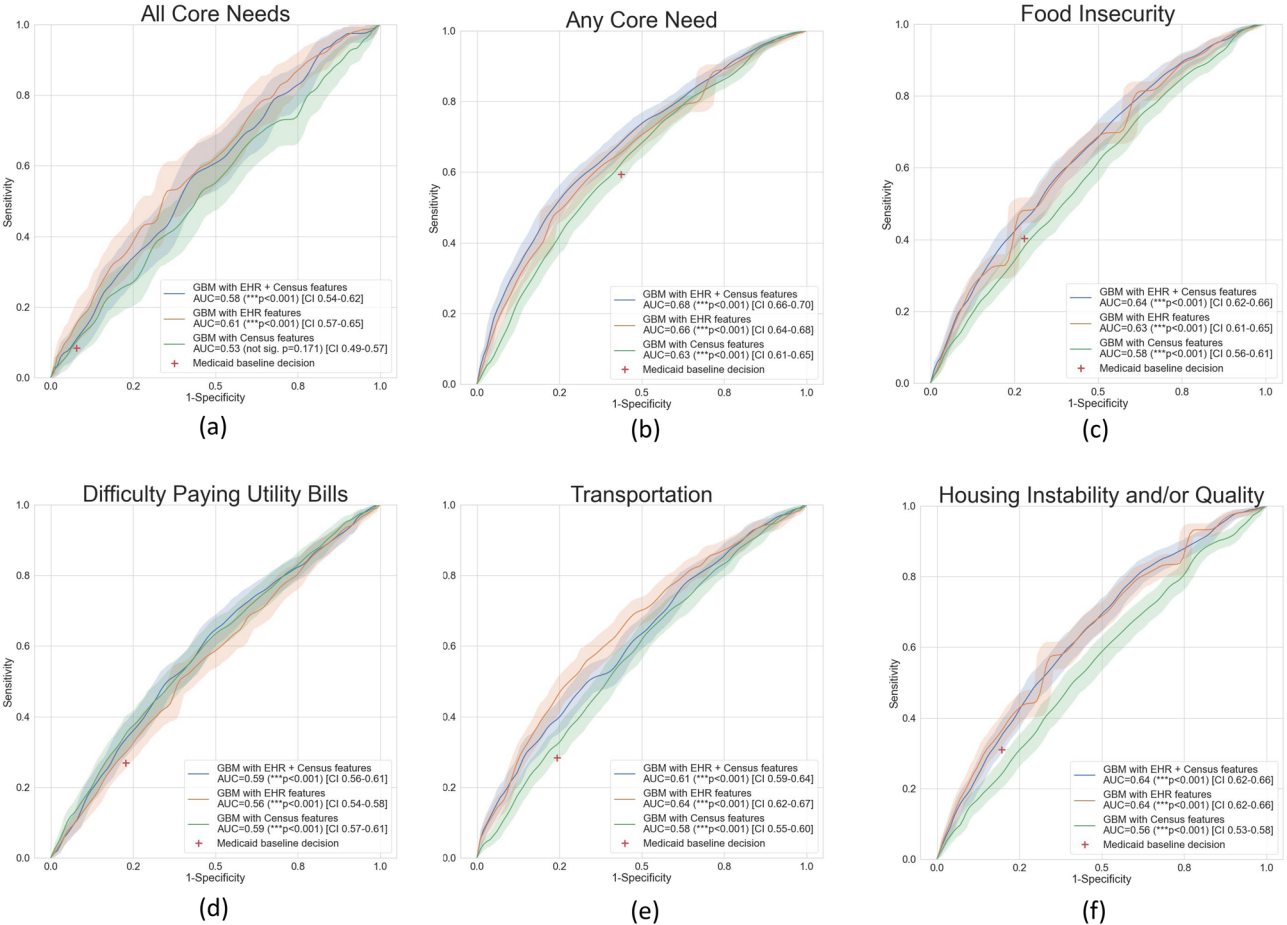
Machine learning model predictive value by HRSN status.

Table 1 summarizes the demographic and HRSN characteristics of the patients included in the final modeling. Patients were primarily female (52.7%), Black or African American (40.6%), single marital status (59.3%), covered by Medicaid (85.4%), and screened at Health System A, a nonprofit, private hospital system (83.8%). Over half of patients (57%) screened positive for at least one HRSN. Food insecurity was the highest frequency need, reported at 39%. Housing, transportation and utility needs were reported with similar frequencies (26–29%). In Fig. 3, we compare and contrast the predictive performance of the ML model trained with the set of features (EHR, Census, or EHR + Census) and the baseline Medicaid status to determine a HRSN. All models trained with EHR and Census features significantly outperformed the baseline Medicaid insurance status to determine presence of a HRSN as shown by the 95% confidence intervals (CI) of the Receiver Operating Characteristic (ROC) curves (shaded areas) when compared to the baseline Medicaid decision (shown as a red cross). When all features were used, AUCs ranged from 0.59 to 0.68. The top performance (AUC = 0.68, CI 0.66–0.70) was achieved by the “any HRSNs” outcome, which is the most useful for patient HRSN screening prioritization. In the majority of experiments, models trained with community-level SDOH features had lower predictive performance than EHR features alone. The only exception was the “Difficulty Paying Utilities” HRSN, where the main drivers for predictive performance were Census features. In order to aid the reproducibility of our findings, all model hyperparameters automatically identified by the Bayesian search are shown in the Supplementary Material.

**Table 1:** Demographic Characteristics and Health-Related Social Needs (HRSNs) of CMS Beneficiaries in the Accountable Health Communities (AHC) Model in the Greater Houston Area, September 2018 to December 2020.

Characteristics	Sample (n = 9800)
	Patients, No. (%)
Age, mean (SD), years	35.5 (26.3)
**Race**	
Black or African American	3978 (40.6)
Other	2857 (29.2)
White	1763 (18.0)
Latin American	612 (6.2)
Hispanic or Latino	337 (3.4)
American Indian or Alaska Native	23 (0.2)
Asian or Pacific Islander	15 (0.2)
Unknown^a^	215 (2.2)
**Marital status**	
Single	5809 (59.3)
Married	1411 (14.4)
Widowed	368 (3.8)
Divorced	365 (3.7)
Separated	67 (0.7)
Life Partner	6 (0.1)
Legally Separated	6 (0.1)
Unknown	1768 (18.0)
**Sex**	
Female	5162 (52.7)
Male	3049 (31.1)
Unknown	1589 (16.2)
**Insurance type**^b^	
Medicaid	8370 (85.4)
Medicare	2231 (22.8)
**Health Related Social Needs (HRSNs)**^b^	
Housing instability and/or quality	2876 (29.3)
Food insecurity	3780 (38.6)
Transportation	2722 (27.8)
Difficulty paying utility bills	2582 (26.3)
Any core need	5588 (57.0)
All core needs	813 (8.3)
**Health System**	
Health System A	8211 (83.8)
Health System B	1108 (11.3)
Health System C	481 (4.9)

^a^Includes "Unknown", "Declined", "Not Answered” responses and records that had no response.

^b^Patients could be in multiple categories so numbers do not sum to total.

## Discussion

We found that the addition of readily available SDOH data at the community-level did not improve performance over data typically available in the EHR for predicting patient social needs status. Of the models, “any HRSN” had the best predictive power at 0.68. Our AUC values were slightly lower than some previous studies^43^, but it is important to note that our outcome (whether the patient reported a HRSN) is different than other currently published studies (referral to a social service), limiting our ability to compare. Use of patient Medicaid insurance status significantly under-predicted social needs status, indicating that use of Medicaid insurance coverage alone is not predictive and we caution providers against using this factor to determine need or who should be screened. Previous studies have shown that patients of lower income status have high rates of social needs, poorer self-rated health, and higher rates of chronic conditions^19^, particularly those with Medicaid and those dually covered by Medicare and Medicaid^57–59^. Given that Medicaid in Texas covers low-income populations and our sample of dually covered beneficiaries was too low to allow splitting between test, training and validation datasets (< 5% of the overall sample), we felt using Medicaid status represented a reasonable baseline hypothesis to apply. What our findings indicate is that the relationship of social needs to insurance status may be more sensitive than previous literature has been able to detect without screening tools. The AHC screening tool recently underwent psychometric testing and was found to have concurrent validity and be sensitive for detecting social needs across a wide swath of patients^60^. When compared to other tools, AHC was more sensitive to detecting certain social needs including housing instability.

Our study adds to the growing literature on the application of machine learning and integration of community SDOH data for use in healthcare settings^39^. Studies to date have found mixed results when adding SDOH data, with some reporting minimal to no improvements in model prediction^43^. Similar to these studies, we found that the addition of SDOH data led to very little improvements to model performance for HRSN status with the exception of difficulty paying utility bills. As other authors have identified, there may be a number of reasons why community-level SDOH are not good predictors of HRSNs. First, while our study included a large population of patients, their geographic locations were clustered into a small number of Census Tracts. The similarity of demographic and SDOH factors resulting from relatively few Census Tracts may have limited discriminatory power^43^. As noted in previous studies^40^, the SDOH factors could be correlated with the patient-level demographics and health status in existing EHR data, therefore, adding limited predictive power in the models from community-level SDOH.

Additionally, we only examined four of the domains of HRSNs, thus it is possible that the SDOH and EHR data might have predictive power in other social need domains such as financial health, social isolation, community safety, and health literacy^61–63^. While the SDOH data from community sources and HRSN screening data measure different constructs, different levels of the associated constructs or different time periods for associated constructs, might impact discriminatory power. For example, difficulty paying utility bills conceptually aligns with SDOH community-level socioeconomic status, particularly income and poverty. The AHC Model survey question asks about difficulty paying bills for the previous 12 months. Whereas, questions from the survey for housing ask about today. Future studies using survey design methods to consider variation in constructs, levels of measurement, and impact of time period assessed are warranted. Lastly, a larger, more geographically and socially diverse sample could be valuable to future modeling efforts to determine if SDOH are truly predictive or not for HRSN status. It is also possible that the high rate of social needs observed in our population limited discriminatory power. This is similar to previously published studies showing high rates of HRSN in ED populations^34^.

Our study offers a number of strengths to the existing literature on the application of machine learning models for predicting HRSNs. To our knowledge this is the first study to directly model HRSN status using publicly available data, EHR data, and individual level HRSN screening data. We utilized data from three large health systems representing patients from the largest medical center in the world. The EHR included both ambulatory and inpatient visit data in addition to patient demographics. We used individual level data on a large number of patients who were screened for HRSN through a universal offer to screen. We used readily available EHR and public SDOH data to model HRSN status making our approach easily replicable by other researchers and health systems. We also compared our findings with Medicaid insurance status as a baseline assumption and potential proxy for HRSN status. Previous studies have found strong associations between Medicaid coverage, social needs, and healthcare utilization and outcomes^64^. Finally, we used state of the art Gradient Boosting Decision Tree ML approaches whose hyperparameters where automatically tuned with Bayesian optimization without using a non-overlapping test set. This allowed for a fully unbiased fine tuning of the algorithm to each HRSN without direct trial and error which could lead to overfitting.

Using community-level SDOH data to predict individual HRSN status collected via screening is prone to limitations and potential biases. First is the risk of ecologic fallacy, where assumptions made about individuals using aggregate-level (area) data yield incorrect results^65^. Despite the value of using such data to predict HRSN status, our study adds to previously published findings that the ecological fallacy may be a limitation to the utility of such efforts.

Second, a potential limitation is the use of individual level HRSN screening data collected via self-report. Self-reported data are prone to bias. Characteristics may differ from those who agreed to answer the HRSNs questions versus those who declined, though our high survey completion rate (~ 45%) lessens this likelihood. Patients might also underreport HRSNs because of social stigma, social desirability bias, or lack of perceived benefit of reporting needs (i.e., access to navigation services^66^).

Lastly, additional limitations relate to the selected SDOH data used in our study. We selected community-level SDOH factors based on previously published studies. However, there is a vast diversity of secondary data available and it is possible that there is an unmeasured and un-modeled SDOH factor, which could improve predictive performance^40^.

There are implications from this study for healthcare providers and institutions. First, targeting those patients with the highest social and health needs could help improve patient health and healthcare utilization. A previous study has shown that social interventions targeting high-utilizing patient populations decreased overall healthcare utilization with more significant effects seen in low–socioeconomic status patients^67^. In terms of hospital utilization, a dose–response relationship has been reported between HRSNs and hospital readmission^68^. This further highlights the need to understand and intervene on high-utilizing populations with social needs.

Second, there is a need to identify how to best screen patients for social needs while reducing clinic burden across healthcare setting types. Machine learning methods can help prioritize patients for HRSN screening while reducing clinic burden^39^. Predicting HRSNs could reduce the need for additional data collection, EHR infrastructure, staff time, and training needed to offer the screening^69^. However, we did not have the ability to screen out any patient group or target people for future intervention without the risk of missing or excluding people. It is difficult to define a threshold for predictive accuracy that would be acceptable. Different accuracy thresholds may be acceptable depending on multiple factors including the specific use case (e.g., prioritizing screening vs. excluding a subpopulation from screening), institutional resources, and other factors. Based on our model prediction and AUC, our results indicate that providers need to continue to use universal offer to screen approaches while more research is conducted on how to best model social needs status and on the clinical and cost effectiveness of social needs screening across healthcare settings^27^. Ideally, a future analysis would apply data from all 28 AHC Model sites in the US coupled with their EHR data to provide a large and geographically diverse enough sample to test the potential predictive power and application of risk modeling for HRSNs.

Third, the integration of SDOH with EHR data has implications for healthcare institutions with the shift to value-based care in the U.S.^39^. The use of community and individual level data could help identify factors associated with social needs to improve healthcare utilization and health outcomes.

We examined the predictive power of HRSN status using community-level SDOH data with individual patient EHR data. We found the addition of SDOH data led to very little improvement in model performance, with the exception of the presence of a utility need. Models trained with EHR and SDOH data performed better than Medicaid insurance status alone. However, screening only these patients identified by the better performing models would miss many patients with HRSNs. Future studies should examine variation of SDOH and EHR data in a geographically broader patient sample to identify possible model enhancements to predict HRSN status and prioritize patients for social interventions.

## Supplementary Information

Supplementary Information 1.

Supplementary Information 2.

## Data Availability

The AHC and EHR datasets generated during and/or analyzed during the current study are not publicly available due to identifying beneficiaries and clinical site information, but are available from the corresponding author on reasonable request.
