# Supplementary material for: Predicting health-related social needs in Medicaid and Medicare populations using machine learning
Source: Sci Rep. 2022 Mar 16;12:4554. doi: 10.1038/s41598-022-08344-4 (PMC8927567; doi:10.1038/s41598-022-08344-4)
Supplement: Supplementary file 1 — Supplementary Information 1. [file 41598_2022_8344_MOESM1_ESM.docx]

## Data Features

We used data from the EHR and US Census Bureau to create the features to be used during the modeling phase. This data included diagnosis, procedure codes, number of ER visits, patient demographics and census data. We used the following features:

- Diagnostics for encounters up to two years prior to the survey date. We used one-hot encoding to represent diagnosis as binary indicators. A sample would have a value of 1 for the columns corresponding to the diagnostics existent for the patient, and 0 for all other columns.
- Procedures for encounters up to two years prior to the survey date. Procedures were represented by the frequency of procedures for each patient, where the columns corresponding to each procedure would have a number corresponding to the count of procedures found for that patient, and 0 for all other columns.
- Number of ED visits reported on the survey. This was reported as a categorical metric, with values ‘ZERO’, ‘ONE’ and ‘TWO_OR_MORE’. We used one-hot encoding to represent this metric.
- Number of ED visits from EHR data. This was calculated as the number of procedures with codes in the range [99281, 99285].
- Patient age (at the time of the survey). This was represented as an integer.
- Census tract. We used one-hot encoding to represent census tract as binary indicators.
- Medicare and Medicaid. Represented as binary indicators. If the patient was enrolled in the corresponding program, the sample would have a value of 1, otherwise it would be 0.
- Census data. All metrics were calculated at the census tract level, with the exception of the metric for Educational Attainment, which was calculated at the county level. We used the following metrics:
  1. Unemployment rate – calculated as a percentage for the census tract.
  2. Poverty level – percentage of families and people whose income in the past 12 months is below the poverty level.
  3. Median household income – calculated as a percentage of the maximum household income for the census tract divided by the maximum household income for all census tract. The maximum value allowed for the household tract is $250,000.
  4. Overcrowding rate – percentage of households in the census tract with more than one occupant per room.
  5. Car ownership – percentage of households in the census tract with no vehicles available.
  6. Home ownership – percentage of housing units in the census tract occupied by the owner.
  7. Disability – percentage of the population of the census tract with a disability.
  8. SNAP benefits – percentage of the households in the census tract receiving food stamps.
  9. Public insurance coverage – percent of population in the census tract covered by insurance.
  10. Uninsured – percent of population in the census tract uninsured.
  11. Social Vulnerability Index (SVI) – index published by the Centers for Disease Control and Prevention (CDC) measuring minority and language status.
  12. Educational Attainment – percent of the population of the county who is 25 years or older with a High School degree or higher.

Most common diagnoses (by count of billing codes) in the population sample:

| **ICD-10 category** | **Category description** | **Number of codes found** |
| --- | --- | --- |
| I10 | Essential (primary) hypertension | 2292 |
| R10 | Abdominal and pelvic pain | 2129 |
| R06 | Abnormalities of breathing | 1808 |
| R07 | Pain in throat and chest | 1800 |
| R94 | Abnormal results of function studies | 1642 |
| Z23 | Inoculations and Vaccinations | 1627 |
| Z3A | Weeks of gestation | 1594 |
| M79 | (Other and unspecified) soft tissue disorders (, not elsewhere classified) | 1588 |
| M25 | (Other) joint disorder (, not elsewhere classified) | 1531 |
| E11 | Type 2 diabetes mellitus | 1361 |
