# Supplementary material for: Predicting health-related social needs in Medicaid and Medicare populations using machine learning
Source: Sci Rep. 2022 Mar 16;12:4554. doi: 10.1038/s41598-022-08344-4 (PMC8927567; doi:10.1038/s41598-022-08344-4)
Supplement: Supplementary file 2 — Supplementary Information 2. [file 41598_2022_8344_MOESM2_ESM.docx]

Supplementary Material/Appendix. A Hyperparameter Optimization

|  | **Max_depth** | **Num_leaves** | **Feature_fraction** | **Subsample** |
| --- | --- | --- | --- | --- |
| **Range** | **[2, 63]** | **[7,1000]** | **[0.1, 1.0]** | **[0.1, 1.0]** |
| **EHR and census data** |  |  |  |  |
| - Housing | 37 | 17 | 0.3969 | 0.3375 |
| - Food | 5 | 7 | 1.0 | 0.4878 |
| - Transportation | 45 | 997 | 0.5559 | 0.4249 |
| - Utilities | 63 | 132 | 0.9983 | 0.9647 |
| - Any needs | 5 | 7 | 0.8560 | 0.4182 |
| - All needs | 59 | 321 | 0.4690 | 0.7031 |
| **EHR data** |  |  |  |  |
| - Housing | 3 | 21 | 0.17412 | 0.1223 |
| - Food | 2 | 97 | 0.2321 | 0.1 |
| - Transportation | 40 | 162 | 0.1030 | 0.6545 |
| - Utilities | 34 | 834 | 0.5238 | 0.8786 |
| - Any needs | 11 | 934 | 0.6885 | 0.1 |
| - All needs | 21 | 952 | 0.8536 | 0.8777 |
| **Census data** |  |  |  |  |
| - Housing | 55 | 420 | 0.9599 | 0.4429 |
| - Food | 5 | 7 | 0.1 | 0.1268 |
| - Transportation | 12 | 194 | 0.7606 | 0.8204 |
| - Utilities | 50 | 7 | 0.1450 | 1.0 |
| - Any needs | 3 | 989 | 0.6011 | 0.4598 |
| - All needs | 59 | 321 | 0.4690 | 0.7031 |
